# Case Report: Chronic Lymphocytic Leukemia With Recurrent Complement-Mediated Thrombotic Microangiopathy and C3 Glomerulonephritis

**DOI:** 10.3389/fmed.2022.813439

**Published:** 2022-02-10

**Authors:** Tiantian Ma, Hui Wang, Tao Su, Suxia Wang

**Affiliations:** ^1^Renal Division, Department of Medicine, Peking University First Hospital, Beijing, China; ^2^Institute of Nephrology, Peking University, Beijing, China; ^3^Key Laboratory of Renal Disease, Ministry of Health of China, Beijing, China; ^4^Key Laboratory of Renal Disease, Ministry of Education, Beijing, China; ^5^Laboratory of Electron Microscopy, Ultrastructural Pathology Center, Peking University First Hospital, Beijing, China

**Keywords:** chronic lymphocytic leukemia/small lymphocytic lymphoma, thrombotic microangiopathy, cryoglobulin, monoclonal IgMκ, C3 glomerulonephritis

## Abstract

Chronic lymphocytic leukemia/small lymphocytic lymphoma (CLL/SLL) is a monoclonal B cell lymphocytosis that produces nephrotoxic monoclonal immunoglobulin (MIg). However, the role of MIg in CLL and how it affects CLL patient survival are still unknown. Here, we report a case of MIg with renal significance (MGRS) associated with CLL. A 59-year-old Chinese woman complaining of abdominal pain, skin purpura, and typical soy-colored urine was admitted to the hospital for investigation. Laboratory tests revealed that she had microangiopathic hemolytic anemia, thrombocytopenia, acute kidney injury (AKI), and hypocomplementemia. She also reported cryoglobulinemia, thrombotic microangiopathy (TMA), and AKI 2 years previously. Peripheral blood smears at that time showed 4% schistocytes, a negative Coombs' test, and elevated lactate dehydrogenase (LDH). Based on a diagnosis of complement-mediated TMA, the patient was treated by plasmapheresis and achieved clinical disease remission. However, the serum hypocomplement 4 and cryoglobulinemia persisted. Further investigation showed elevated B lymphocytes and monoclonal serum IgMκ; however, the cryoprecipitate contained monoclonal IgMκ and polyclonal IgG, as well as immunoglobulins κ and λ. After plasmapheresis, her LDH, platelets, and complement 3 (C3) levels returned to normal. Biopsies of the bone marrow and an enlarged subclavicular lymph node revealed CLL/SLL. Renal pathological findings indicated significant arteriolar endothelial cells myxoid edema and glomerular endothelial cells swelling, however no thromboli, cryoglobulin formation and vasculitis were observed. We also found mild mesangial proliferative C3 glomerulonephritis and renal interstitial CLL cells infiltration. Collectively, these clinical and pathological manifestations were attributed to monoclonal IgMκ, which triggered C3 activation. MGRS associated with CLL was finally confirmed. Six cycles of rituximab, cyclophosphamide, verodoxin, and dexamethasone therapy were administered, after which she received ibrutinib. The patient experienced disease remission, and her serum C4 level returned to normal. Cryoglobulin and IgMκ were not detected. This is a special presentation of CLL/SLL with monoclonal IgMκ, which is a type of MGRS. Activation of the complement system by MIg led to TMA with C3 glomerulonephritis. Treatment for TMA and CLL/SLL should be initiated in a timely manner to improve patient prognosis.

## Introduction

Chronic lymphocytic leukemia/small lymphocytic lymphoma (CLL/SLL) is rare in the Asian population (1). CLL/SLL is a monoclonal B cell lymphocytosis, a diagnosis that is equivalent to monoclonal gammopathy of undetermined significance for clones of the CLL lineage, and produces nephrotoxic monoclonal immunoglobulin (MIg). CLL is characterized by the clonal expansion of CD5^+^CD19^+^CD23^+^ surface immunoglobulin^+^CD20^dim^ B cells in the blood, marrow, and secondary lymphoid tissues. The occurrence of kidney diseases associated with this small, low-grade clonal disorder in the absence of symptomatic CLL is rare but becoming more common (2, 3). Disease severity and clinical presentation diversity are closely related to the specificity of B cell clones and the serum concentration of M-spike in CLL-monoclonal immunoglobulinemia with renal significance (MGRS). However, the role of Ig paraprotein in CLL and how serum Ig paraprotein affects CLL patient survival are still unknown. Further, there is also little evidence on the long-term prognosis of patients treated with chemotherapeutics.

## Case Description

**Figure 1:**
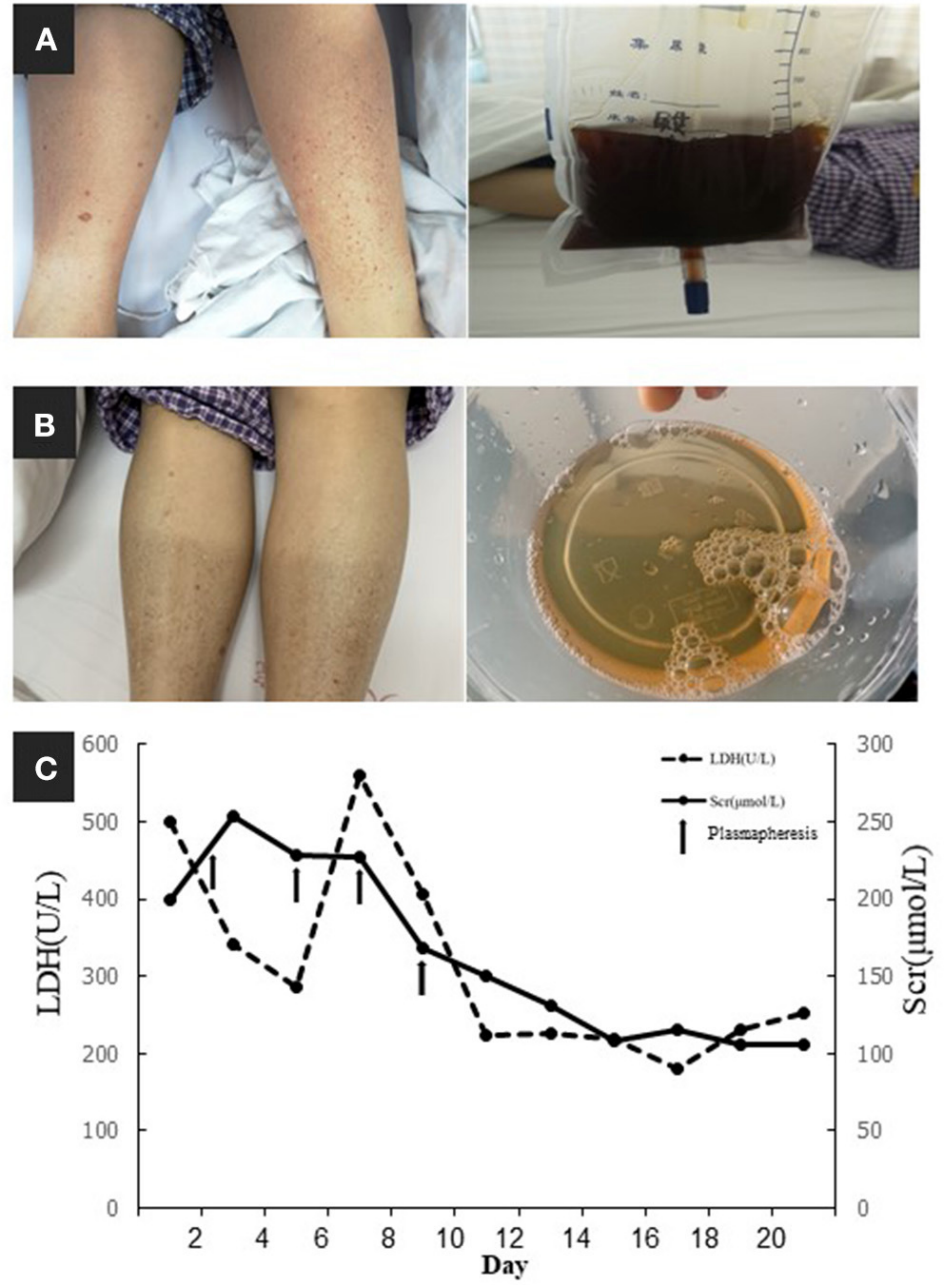
**(A)** Skin purpura and typical soy-colored urine. **(B)** The remission of purpura and hematuria. **(C)** Timeline of serum creatinine (Scr) and lactate dehydrogenase (LDH) and their relationships with plasmapheresis (arrows indicated plasmapheresis).

On April 28, 2019, a 59-year-old Chinese woman was admitted to our hospital because of abdominal pain, skin purpura, and typical soy-colored urine (Figures 1A,B) when the temperature dropped suddenly. She experienced a rapid decrease in urine volume and developed oliguria over the following 5 days. The patient was diagnosed with thrombotic microangiopathy (TMA) based on the signs of microangiopathic hemolytic anemia and thrombocytopenia, as follows: hemoglobin, 9.5 g/dL (11.5–15.0 g/dL); platelet count, 94 × 10^9^/L (125–350 × 10^9^/L); and lactate dehydrogenase (LDH) 284 U/L (100–240 U/L). The direct Coombs' test and antiplatelet antibody test results were negative. Peripheral blood lymphocyte count was slightly elevated at 3.34 × 10^9^/L (1.1–3.2 × 10^9^/L) and blood smears showed 0.3% schistocytes. Further, serum creatinine peaked at 187 μmol/L (44–133 μmol/L), and the complement 3 (C3, 0.372 g/L, range 0.6–1.5 g/L) and C4 (below the detection limit) concentrations were extremely low. Urinalysis indicated proteinuria (2+) and hematuria (15–20 red blood cells per high-power field). The patient had experienced cryoglobulinemia, typical TMA with 4% schistocytes, a negative Coombs' test, and elevated LDH and acute kidney injury (AKI) 2 years previously. A diagnosis of complement-mediated TMA was made, and the patient undertook plasmapheresis and achieved clinical disease remission, except for persistently low serum C4 and cryoglobulinemia in the remission stage.

**Figure 2:**
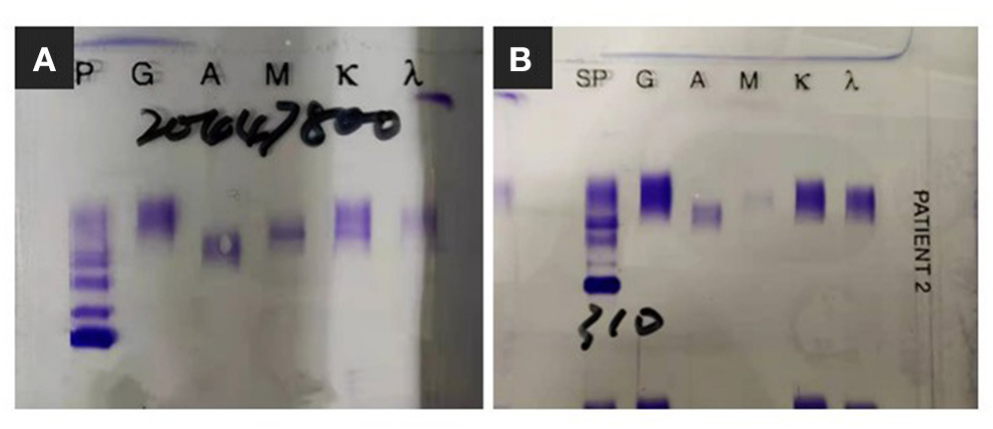
Immunofixation electrophoresis findings. **(A)** Monoclonal IgMκ in the serum. **(B)** Monoclonal band was not detected since clinical complete remission.

For further investigation of the recurrent TMA, we assessed ADAMTS13 (A Disintegrin and Metalloprotease with a ThromboSpondin type 1 motif, member 13) activity and complement factor H concentration, both of which were normal. No anti-factor H antibodies or C3 nephritis factors were detected. The urine test and free hemoglobin test results were negative. Other serological tests for antinuclear antibodies, anti-phospholipid antibodies, hepatitis C virus, and hepatitis B virus were negative. Immunofixation electrophoresis detected monoclonal IgMκ in the serum (Figure 2A); however, the cryoprecipitate contained monoclonal IgMκ and polyclonal IgG, as well as immunoglobulin light chains κ and λ. Pathological analysis of biopsy samples from the bone marrow and an enlarged subclavicular lymph node revealed CLL/SLL. The result was consistent with the finding of lymphocytosis in peripheral blood cell analysis (WBC, 9.72 × 10^9^/L [3.5–9.5 × 10^9^/L]; B-cell count, 1431.46/μL [80–616/μL]). The patient again underwent four courses of plasmapheresis. Hemolysis and thrombocytopenia were relieved rapidly with simultaneous improvement in kidney function, but she experienced massive proteinuria (Figure 1C).

**Figure 3:**
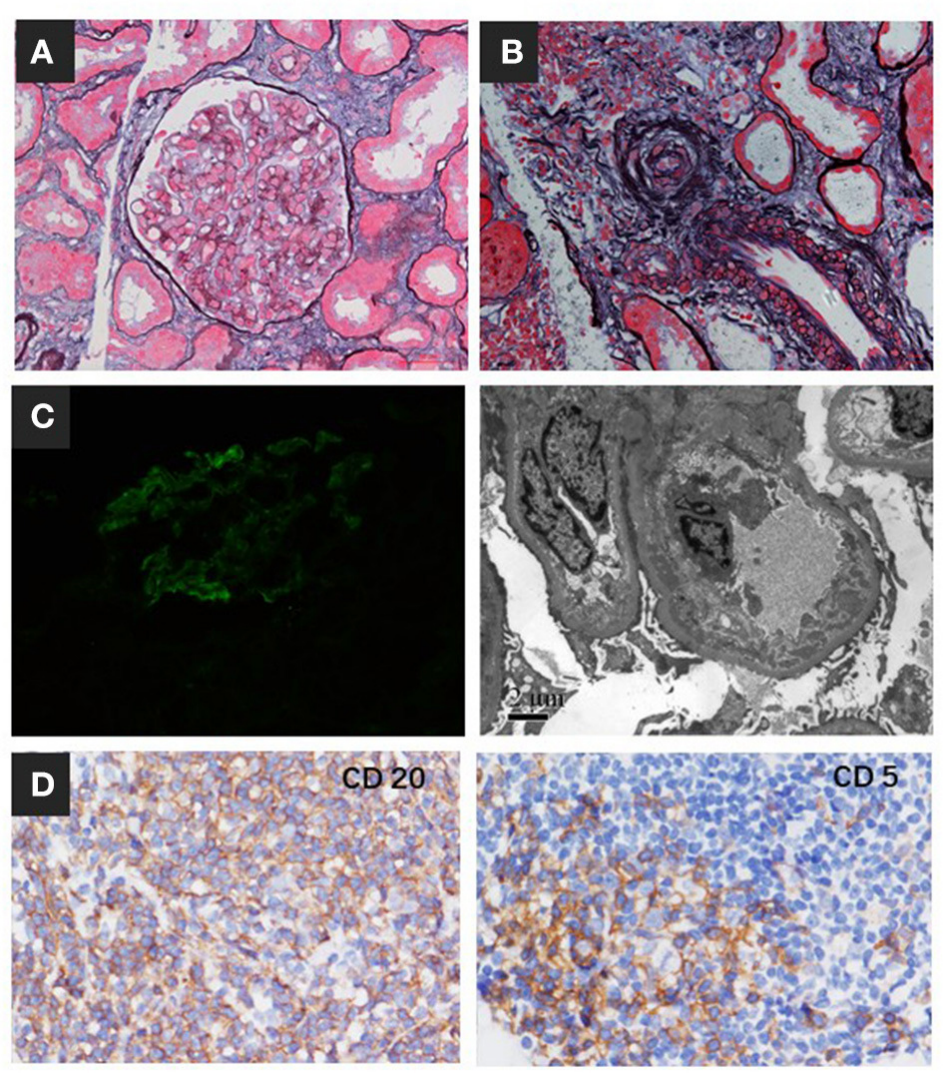
Renal biopsy findings. **(A)** The glomerulus showed segmental endothelial cell proliferation and swelling (periodic acid-silver methenamine, Masson staining, ×400). **(B)** Interlobular renal arteries showed intimal swelling and contained mainly lucent amorphous material with a mucoid appearance, which we classified as mucoid intimal hyperplasia. **(C)** Immunofluorescence staining showed C3 deposits in the mesangial area. The expansion of the lucent subendothelial zone of the glomerular capillary had dense deposition in the subendothelial and mesangial area by EM. **(D)** Immunohistochemistry showed monotypic lymphocytes infiltrating the renal interstitium that were positive for CD20 and CD5.

Based on these results, a diagnosis of CLL-associated MGRS was suspected. After the remission of thrombocytopenia, renal biopsy was performed. Immunofluorescent microscopy of the biopsy sample showed only mild C3-dominant deposits in the mesangial area, and immunohistochemical staining showed no “masked” immune deposits on the glomerular capillary loops. Light microscopy revealed TMA-like microangiopathic lesions. The interlobular renal arteries had intimal swelling that was sparsely cellular and contained mainly lucent amorphous material with a mucoid appearance (Figures 3A,B), which we classified as mucoid intimal hyperplasia. Electron microscopy also showed expansion of a lucent subendothelial zone of the glomerular capillary with dense deposits in the subendothelial and mesangial areas (Figure 3C). In addition, monotypic small lymphocytes infiltrated the renal interstitium, which expanded the interstitium at the expense of the tubular structures. These monotypic lymphocytes were positive for CD20, CD5, and BCL2 and negative for cyclin D1, CD10, CD23, CD138, and CD68 (Figure 3D). We speculated that cold weather triggered the formation of massive monoclonal cryoglobulin precipitation, which strongly activated the complement system. Complement mediated TMA was confirmed with a concurrent mesangial proliferative C3 glomerulonephritis (C3GN) and renal interstitial CLL infiltration. The cells in the lymph node showed features of SLL and had mutated variable immunoglobulin heavy chain variable region genes, a marker correlated with a more favorable prognosis. This information in combination with the immunohistochemical staining results of CD3, CD20, PAX5, BCL2, CD5, CD23, CD21, and CD43 indicated CLL/SLL stage II according to the Rai staging system.

**Figure 4:**
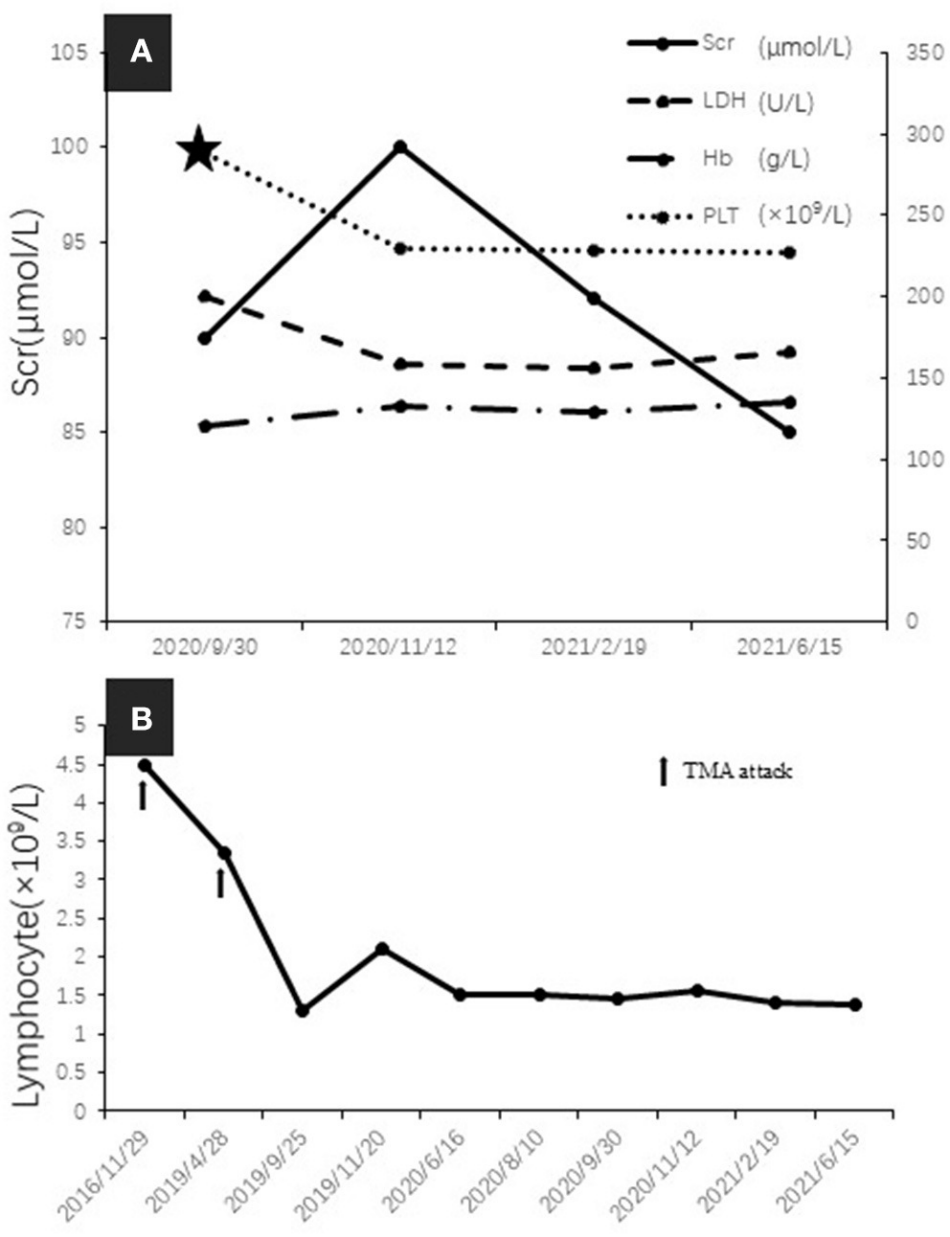
**(A)** Serum creatinine (Scr), lactate dehydrogenase (LDH), hemoglobin, and platelet count over time during remission (five-pointed star indicated the commencing date of Ibrutinib treatment). **(B)** The change of peripheral blood lymphocyte count (arrows indicated TMA attack).

Since bendamustine was not available in our hospital, the initial chemotherapy regimen was rituximab, cyclophosphamide, verodoxin, and dexamethasone (R-COP). Six cycles of R-COP were administered from September 19, 2019 to September 30, 2020, and then the patient was administered ibrutinib. She responded well and is currently in complete remission, without evidence of monoclonal immunoglobulin (Figure 2B) and cryoglobulinemia, with serum C4 and lymphocyte count in the normal range (Figures 4A,B).

## Discussion

Kidney disease in patients with CLL/SLL is under-recognized, as CLL/SLL usually has an indolent course and kidney biopsy is seldom performed. However, in a study by the Mayo Clinic, renal insufficiency was present in 7.5% of patients with CLL at the time of diagnosis, and a further 16.2% developed renal insufficiency during the course of the disease. Kidney disease was associated with worse patient outcomes in their cohort (4). Here, we describe the case of a 59-year-old Chinese woman who initially presented with AKI caused by TMA. In the investigation to determine the underlying causes of the TMA, type II cryoglobulinemia was detected, which was composed of monoclonal IgM and κ, polyclonal IgG, and immunoglobulins κ and λ. Pathological examination of the bone marrow and enlarged supraclavicular lymph node indicated CLL/SLL. We observed concurrent swelling of interlobular arterioles intima and glomerular endothelial cells and C3GN with CLL cells infiltrating the renal interstitium. Both were speculated to be mediated by MIgs produced by the B-cell clone.

The presence of minimal B-cell lymphoma clones and cytogenetic features that can be detected by peripheral blood flow cytometry explain the diverse clinical phenotypes of CLL/SLL and are helpful in disease management. Kidney disease induced by low-grade CLL is classified according to the new International Kidney and Monoclonal Gammopathy Research Group consensus definition (5). In patients who develop renal lesions that lead to life-threatening complications because of CLL, therapeutic intervention should be initiated immediately to prevent further damage.

It has been reported that CLL in Asian populations has different features than CLL in populations predominately of European descent (1, 6). Atypical morphologic and immunologic features have been described in Asian patients with CLL. Ig paraprotein has been reported in 19.8–20.3% of Chinese CLL cohorts at diagnosis (7, 8), which is much higher than the 11–14.8% reported in European CLL cohorts (9, 10). Autoimmune phenomena may precede or accompany lymphoid malignancies.

The frequency of MIg in CLL is second only to that in Waldenström macroglobulinemia among lymphoid neoplasms (11, 12). IgM paraprotein is detected in 4.8–4.9% of CLLs (8, 10) and is significantly associated with a more advanced Binet/Rai stage and del(17p)/*TP53* mutations. In addition, IgM paraproteinemia is strongly related to poorer overall survival (13). Our patient had low-grade CLL/SLL with kidney biopsy-proven microangiopathy presenting with critical AKI. Fortunately, she responded well to plasmapheresis, anti-CD20 therapy, and Bruton's tyrosine kinase inhibitors.

MIg may cause kidney injury directly through glomerular deposition or indirectly through dysregulation of the alternative complement pathway, resulting in TMA (14) or C3GN (14, 15). The occurrence of TMA in CLL usually indicates circulating MIg. In the report by Ravindran et al. (16), paraprotein was detected in 13.7% of 148 TMA patients, including 15 (10.1%) patients with MGRS, 1 (0.7%) with MM, 2 (1.4%) with POEMS, and 1 (0.7%) with T-cell lymphocytic leukemia. During the course of TMA treatment, our patient manifested corresponding changes in C3 concentration. This suggested that the complement system was activated by cryoglobulin, leading to dysregulation of the alternative pathway, which played an important role in the pathogenesis of TMA. In addition, there was no evidence supporting TTP, malignant hypertension, toxic drugs, infections, or autoimmune diseases in our patient.

C3GN is another rare type of MGRS resulting from the dysregulation of the alternative complement pathway. The association of C3GN with monoclonal gammopathy has been previously reported (16–18). The hallmark of the disease is the presence of C3 on immunofluorescence microscopy with minimal or no Ig. Acquired abnormalities include antibodies, complement-regulating proteins, or both, such as the presence of C3 nephritic factor and antibodies for complement factor H, factor B, and cofactors (14, 15). Zand et al. reported on 10 patients with C3GN with associated monoclonal gammopathy in the largest case series to date. Bone marrow biopsy revealed monoclonal gammopathy of undetermined significance in 5 patients and CLL in 1 patient (18). Recent studies have focused on the link between MIg and dysregulation of the alternative complement pathway. It is assumed that MIg could act as an antibody to complement fragments such as C3 convertase or CFH, resulting in the uncontrolled activation of the alternative complement pathway. Moreover, monoclonal lambda light chains can activate the alternative pathway by directly interacting with factor H (19) or inhibiting factor H by binding to its third short consensus repeat domain (20).

The persistence of extremely low C4 is another phenomenon of immune disorders, indicating activation of the classical complement pathway. This decrease in C4 is also speculated to be mediated by a massive polyclonal immunoglobulin. Cold agglutinin is a cold-sensitive antibody. This patient experienced severe extravascular hemolysis during disease relapse, which might have resulted from massive erythrocyte aggregation and destruction at low temperatures. Cold agglutinin formation was suspected because cryoglobulin can cause skin vasculitis but does not interact with red blood cells. Unfortunately, we could not measure cold agglutinin in our hospital because of the lack of detection reagents. Moreover, our study had another limitation. The patient was reluctant to undergo genetic testing because of the high cost and denied having a family history of similar illness. Mutations in complement regulatory proteins such as complement factor H-related protein 5 serve as the significant genetic basis for the pathogenesis of TMA. It is worth clarifying.

## Conclusion

In conclusion, this study reports a special presentation of CLL/SLL with monoclonal IgMκ, which is a type of MGRS. Activation of the complement system by these MIgs led to TMA and C3GN. Treatment for TMA and CLL/SLL should be initiated in a timely manner to ensure good patient prognosis.

## Data Availability Statement

The original contributions presented in the study are included in the article/supplementary material, further inquiries can be directed to the corresponding author.

## Ethics Statement

The studies involving human participants were reviewed and approved by Peking University First Hospital, approval number: 2017[1333]. The patients/participants provided their written informed consent to participate in this study. Written informed consent was obtained from the individual(s) for the publication of any potentially identifiable images or data included in this article.

## Author Contributions

TM and TS contributed to patient diagnosis, management, and clinical data analysis. HW and SW contributed to the pathological diagnosis and took and edited pathological images. TM, HW, and TS drafted the manuscript and contributed to data analysis, interpretation, and intellectual content of critical importance to the work described. All authors contributed to the article, revised the manuscript, and approved the submitted version.

## Funding

This research was supported by a grant from the National Science and Technology Major Projects for Major New Drugs Innovation and Development (2017ZX09304028).

## Conflict of Interest

The authors declare that the research was conducted in the absence of any commercial or financial relationships that could be construed as a potential conflict of interest.

## Publisher's Note

All claims expressed in this article are solely those of the authors and do not necessarily represent those of their affiliated organizations, or those of the publisher, the editors and the reviewers. Any product that may be evaluated in this article, or claim that may be made by its manufacturer, is not guaranteed or endorsed by the publisher.

## Abbreviations

CLL: chronic lymphocytic leukemia
SLL: small lymphocytic leukemia
MGRS: monoclonal immunoglobulinemia with renal significance
TMA: thrombotic microangiopathy
C3GN: C3 glomerulonephritis
MIg: monoclonal immunoglobulin
EM: electronic microscopy.
